# The Fumarprotocetraric Acid Inhibits Tau Covalently, Avoiding Cytotoxicity of Aggregates in Cells

**DOI:** 10.3390/molecules26123760

**Published:** 2021-06-21

**Authors:** Camila González, Constanza Cartagena, Leonardo Caballero, Francisco Melo, Carlos Areche, Alberto Cornejo

**Affiliations:** 1 Escuela de Tecnología Médica, Facultad de Medicina, Universidad Andres Bello, Santiago 8370071, Chile; camilagonzalez92@hotmail.cl (C.G.); coni.cartagenap@gmail.com (C.C.); 2 Departamento de Física, Center for Soft Matter Research, SMAT-C, Usach, Avenida Ecuador, Estación Central, Santiago 9170124, Chile; leonardo.caballero@usach.cl (L.C.); francisco.melo@usach.cl (F.M.); 3 Departamento de Química, Facultad de Ciencias, Universidad de Chile, Las Palmeras 3425, Ñuñoa, Santiago 7800003, Chile; areche@uchile.cl

**Keywords:** lichens, tauopathies, α,β carbonyl group, inhibitors, aggregates, cytotoxicity

## Abstract

Neurodegenerative disorders, including Tauopathies that involve tau protein, base their pathological mechanism on forming proteinaceous aggregates, which has a deleterious effect on cells triggering an inflammatory response. Moreover, tau inhibitors can exert their mechanism of action through noncovalent and covalent interactions. Thus, Michael’s addition appears as a feasible type of interaction involving an α, β unsaturated carbonyl moiety to avoid pathological confirmation and further cytotoxicity. Moreover, we isolated three compounds from Antarctic lichens *Cladonia cariosa* and *Himantormia lugubris*: protolichesterinic acid (**1**), fumarprotocetraric acid (**2**), and lichesterinic acid (**3**). The maleimide cysteine labeling assay showed that compounds **1**, **2**, and **3** inhibit at 50 µM, but compounds **2** and **3** are statistically significant. Based on its inhibition capacity, we decided to test compound **2** further. Thus, our results suggest that compound **2** remodel soluble oligomers and diminish β sheet content, as demonstrated through ThT experiments. Hence, we added externally treated oligomers with compound **2** to demonstrate that they are harmless in cell culture. First, the morphology of cells in the presence of aggregates does not suffer evident changes compared to the control. Additionally, the externally added aggregates do not provoke a substantial LDH release compared to the control, indicating that treated oligomers do not provoke membrane damage in cell culture compared with aggregates alone. Thus, in the present work, we demonstrated that Michael’s acceptors found in lichens could serve as a scaffold to explore different mechanisms of action to turn tau aggregates into harmless species.

## 1. Introduction

Alzheimer’s disease (AD) is the most prevalent form of dementia [1], involving beta-amyloid (Aβ) and microtubule-associated protein tau. The protein depositions are characterized by plaques and neurofibrillary tangles, respectively (NFT) [2]. Tau protein participates in axonal transport and microtubule stability; however, several stressful factors can provoke it to detach from microtubules and form aggregates in soma and dendrites neuron cells [3]. Tau pathology involving the microtubule-binding domain (4R) is associated with PSP (progressive supranuclear palsy), CBD (corticobasal degeneration), AGD (argyrophilic grain disease), CTE (chronic traumatic encephalopathy), and GGT (globular glial tauopathy); tau aggregates can be found in the medial temporal lobe, cortex, basal ganglia, subthalamic nucleus, and substantia nigra [4]. Moreover, tau protein has two fibril-forming motifs, ^275^VQIINK^280^ and ^306^VQIVYK^311^. These two motifs are within the microtubule-binding domain (4R) of tau and are prone to form a cross β structure [5]. Moreover, two cysteines have a central role in polymerization as well, and the first step of aggregation occurs by forming a covalent bond between cysteine residues to form soluble oligomers [5].

Interestingly, the first stage of oligomer formation can find a mixture of a random coil and β sheet structure. However, as long the tau polymerization occurs, mature oligomers are formed whose content is a majority of β sheet content [6]. These oligomers’ structures are detected at the prefrontal cortex in Braak stage I [7]. Moreover, the pathological hallmark of AD is extracellular deposits of β-amyloid (Aβ) and intracellular inclusions of tau protein [8]. Thus, these neuropathological features have strongly influenced therapeutic strategies, whose primary focus is Aβ. The amyloid hypothesis relies on the fact that Aβ accelerates the NFT formation and neuronal death in the neocortex [9]. However, all these approaches focused on Aβ, including clinical trials, have failed since there are no cognitive improvements in patients with AD [10].

Additionally, tau is related to FTD linked to chromosome 17, where the neuronal loss occurs in the absence of Aβ [11]. Thus, considering that tau correlates better with cognitive impairment and dementia symptoms, drug discovery strategies focus on tau [12]. Moreover, compounds such as cinnamaldehyde or anthraquinone can interact with tau by inhibiting cysteine interactions, promoting incompetent aggregate forms [13,14]. Here, we present the activities of three lichen compounds capable of inhibiting tau aggregation, and the aggregates treated with compound **2** can prevent cell membrane damage and further LDH leaking.

## 2. Results

### 2.1. Lichens Compounds Inhibit the Progression of Tau Aggregation through the Interaction with Cysteines

**Figure 1:**
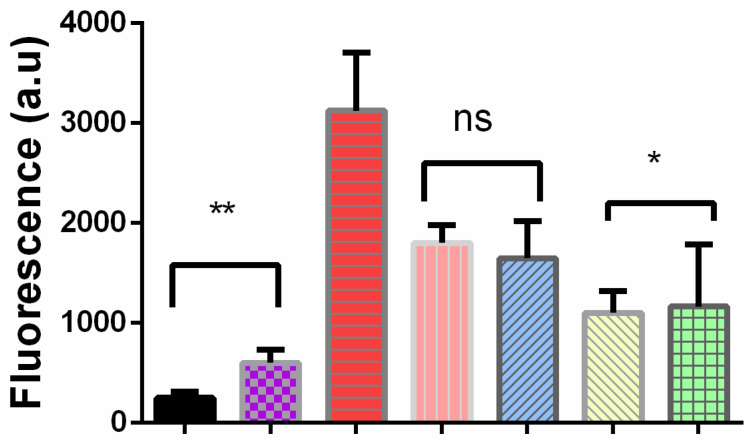
Maleimide cysteine labeling inhibition through covalent interactions of compounds **1**,**2**,**3**. Data were analyzed through one way-ANOVA and Dunnett’s multiple comparison test (*p* < 0.05). Non-significant (ns), significant (*), (**). The black, purple, red, pink, blue, yellow, and green bars represent sodium acetate 100 mM; tau: heparin (4:1) plus Tris(2-carboxyethyl) phosphine (TCEP); tau: heparin (1:4) plus Tris(2-carboxyethyl) phosphine (TCEP); DMSO (oxidizing agent); compound **1**; compound **2**; compound **3**, respectively. The experiments were done in triplicate.

The lichens compound isolated (Figures S1 and S2), tested as tau cysteine inhibitors through the cysteine’s maleimide labeling interaction, represents the interaction of α, β unsaturated carbon with cysteine. The maleimide labeling allows monitoring the tau aggregation based on the cysteine–cysteine interaction. Thus, we induced tau aggregation with heparin in a proportion of 10 µM: 40 µM (1:4) for 4 h. As observed in Figure 1, the control aggregation promotes a strong aggregation in the presence of heparin and Tris(2-carboxyethyl) phosphine (TCEP). In the presence of compounds, the aggregation diminishes, suggesting that all compounds can interact with cysteine, but we found significant differences only in compounds **2** and **3**. As a control, we used dimethyl sulfoxide (DMSO) as an oxidizing agent capable of interacting with cysteine, as shown in Figure 1.

**Figure 2:**
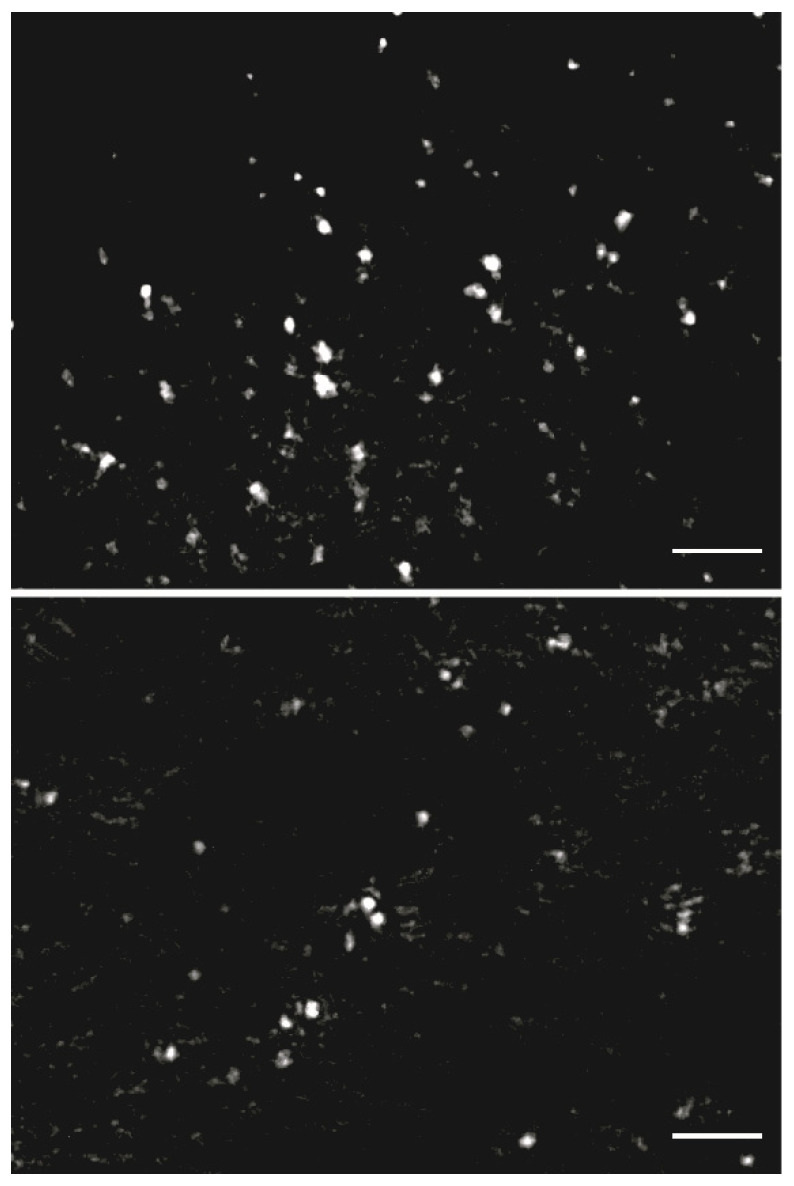
Total internal reflection fluorescence microscopy (TIRFM). Upper panel, TIRFM phase of tau aggregates. Lower panel, TIRFM tau aggregates images treated with compound **2**: bars 20 µm. Images were analyzed through FIJI.

Furthermore, total internal reflection fluorescence microscopy (TIRFM) selectively illuminates the fluorophore linked covalently to cysteine. The control aggregation image (Figure 2, upper panel) showed several spots indicating that aggregations under the induction of heparin occur massively at 48 h. Then, TIRFM images of aggregates in the presence of compound **2** showed fewer illuminated elements, suggesting that compound **2** can interfere with cysteine interactions that promote further assembly of soluble oligomers (Figure 2, lower panel). Moreover, we quantified the illuminated elements in both samples (Figure S3); we found fewer illuminated aggregates in the sample treated with **2**, indicating the interference of compound **2** with aggregation based on the interaction of cysteine. Based on the structure of compound **2**, it is feasible to infer that the interaction of inhibition is driven under the action of the α, β unsaturated carbonyl group.

### 2.2. The Compound **2** Diminished β Sheet Content and Remodeled Aggregates Morphology 

**Figure 3:**
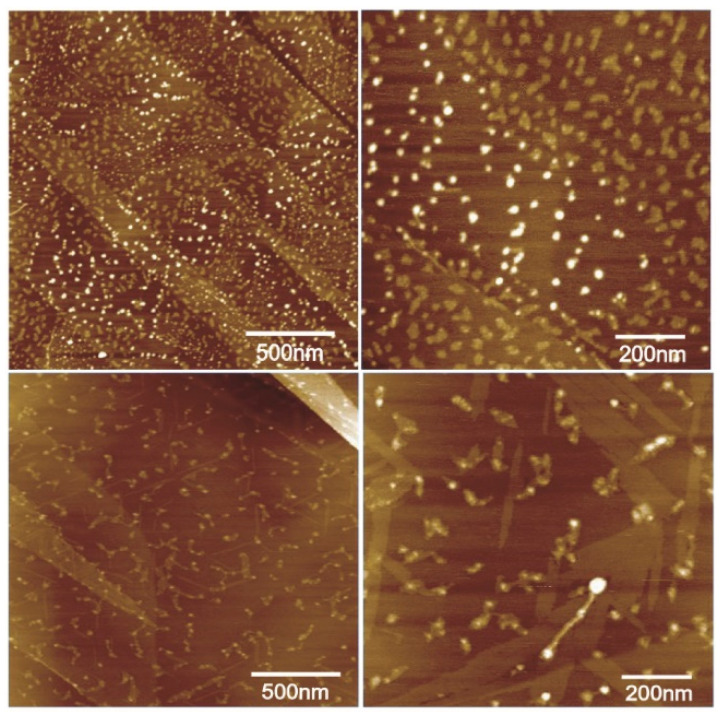
Atomic force images of tau aggregates. Upper panel, phase images of aggregation control. Lower panel, tau aggregates treated with compound **2**: bars 500 and 200 nm, respectively.

The Thioflavin T assay showed that after 48 h of aggregation under heparin induction, we observed the highest amount of β sheet content, as depicted in Figure S4. However, once we incubated compound **2**, the amount of β-sheet content was reduced, as depicted in Figure S4. Additionally, the morphology observed in samples over HOPG of tau aggregates resembles the formation of oligomers and fibrils, as observed in Figure 3.

Figure S5 represents the quantification of aggregates showing heights of 2.5 nm and a length of aggregates from 10 to 100 nm. In the presence of compound **2**, the morphology of aggregates is affected, showing fewer aggregates (Figure 3) with a height of less than 2 nm and length varying from 20 nm to 50 nm (Figure S5).

### 2.3. Cells Incubated with Treated Oligomers by Compound **2** Prevent Morphological Changes

**Figure 4:**
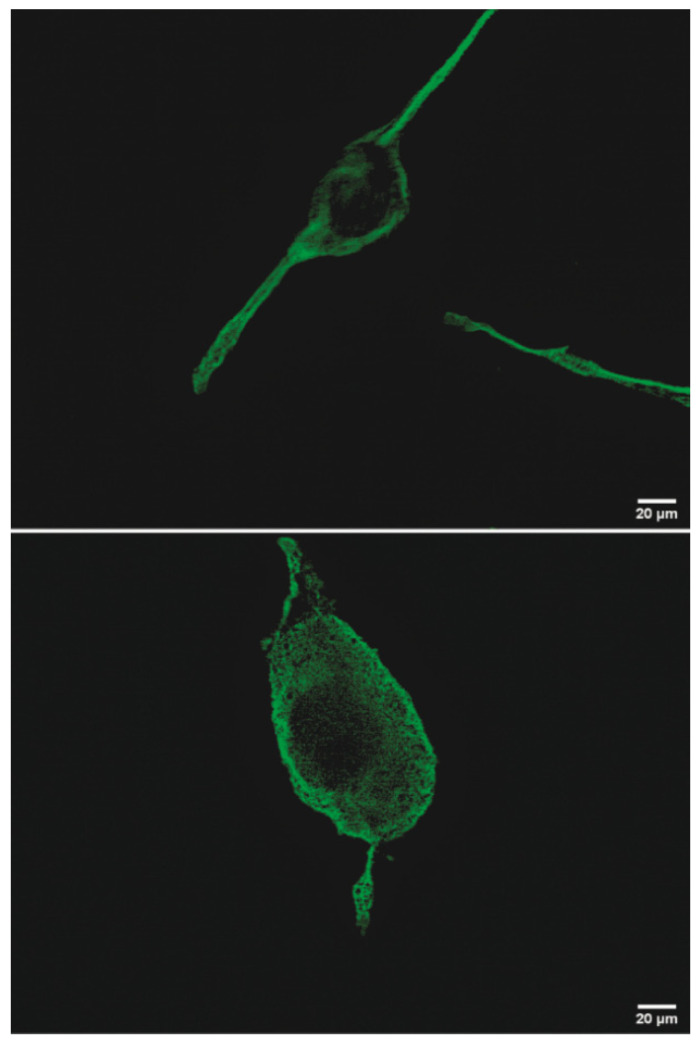
Upper panel, immunofluorescence of N2a cells induced neurite-like control (monomer). Lower panel, immunofluorescence of N2a cells incubated with tau aggregates alone: bars 20 µm.

**Figure 5:**
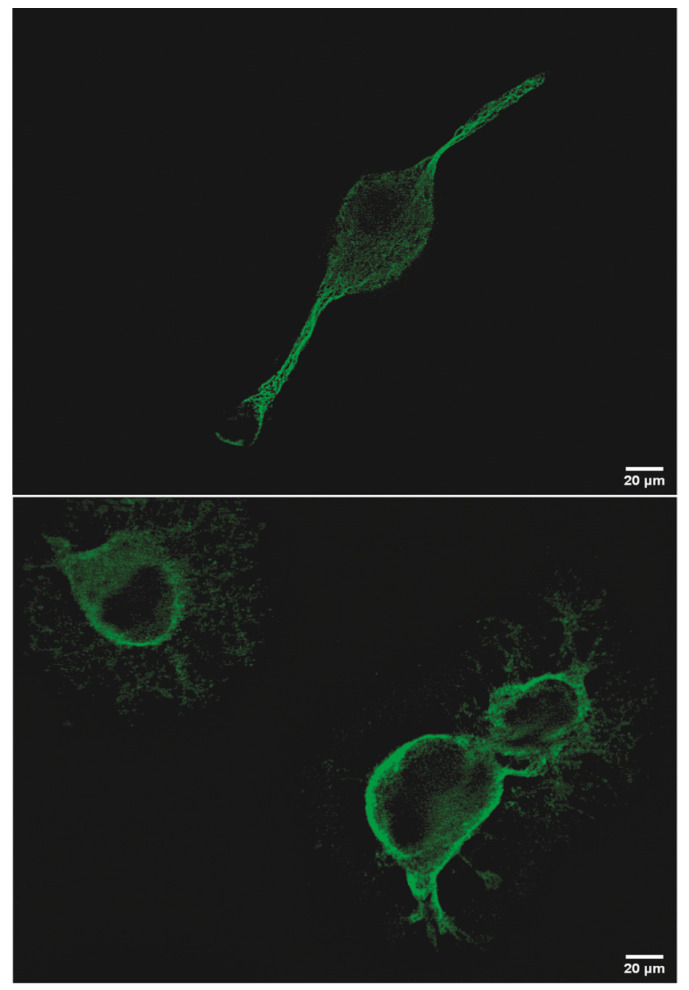
Upper panel, immunofluorescence of N2a cells incubated for 24 h with oligomers treated with compound **2**. Lower panel, immunofluorescence of N2a cells incubated with tau aggregates alone: bars 20 µm.

To resemble the neurite-like outgrowth, we cultured N2a cells in the presence of retinoic acid 10 µM for four days. Then, 100 nM of tau aggregates treated with compound **2** or aggregates alone were incubated for 24 h in cultured cells. Thus, we observed a neurite-like induced extension that resembles neuritogenesis (Figure 4, upper panel). Hence, the neurite-like outgrowth involves cytoskeletal proteins, such as microtubules conformed by α, β tubulin dimers, depicted in Figure 4 (upper panel). However, in the cells incubated in the presence of oligomers, the morphology of the cells is affected, and the cells suffered the shortening of the neurite-like structure, suggesting a harmless effect provoked through the external oligomers added to the culture medium, as observed in the lower panel of Figure 4. Then, oligomers inhibited through a covalent bond were incubated in cell culture, observing neither changes in the morphology nor retractions in the neurite-like outgrowth, as seen in Figure 5 (upper panel). Moreover, in cells cultured with nontreated oligomers, the morphology is affected, and the neurite retraction also occurs, as seen in Figure 5 (lower panel), suggesting a deleterious effect of tau aggregates.

### 2.4. Cells Cultured in the Presence of External Oligomers Provoke Membrane Damage

**Figure 6:**
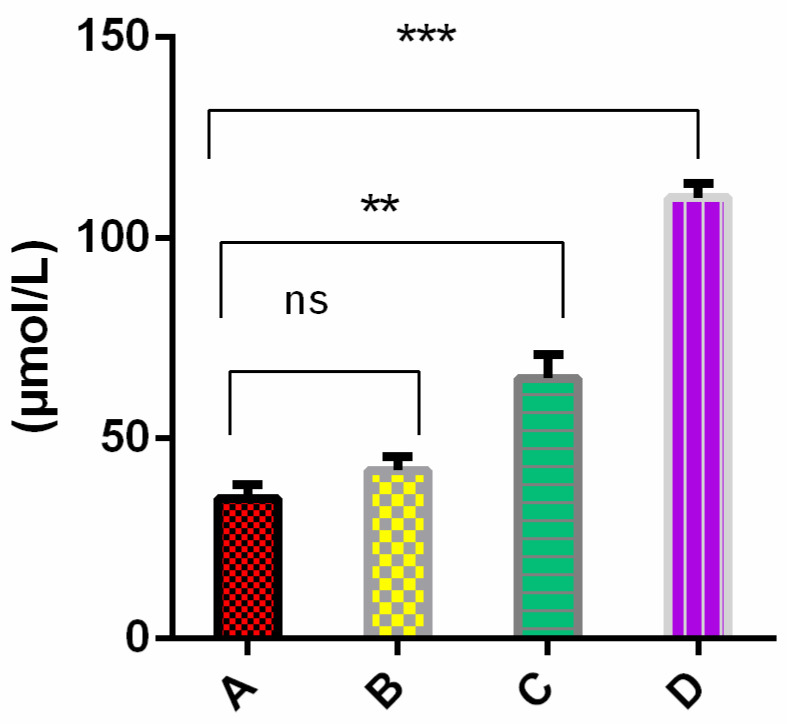
Lactate dehydrogenase assay of N2a cell incubated with tau aggregates treated with **2** or tau aggregates alone for 24 h, then the supernatant tested. Data are represented as mean and SD and analyzed through one-way ANOVA and Dunnett’s multiple comparison test (*p* < 0.05). Non-significant (ns), significant (**), (***). The red, yellow, green, and purple bars represent control (monomers), treated aggregates, aggregates alone, and lysis buffer, respectively.

As seen in the immunofluorescence images, the incubated oligomers in cell cultures remodel their morphology, provoking neurite-like retraction, suggesting the involvement of cytotoxic events. Thus, we decided to test LDH leaking to detect membrane damage since it correlates well with membrane damage. Thus, after incubation for 24 h with either treated oligomers by compound **2** or oligomers alone, we recovered the supernatant from detecting LDH through a colorimetric assay. Therefore, in Figure 6, the control (monomer) and the oligomers treated with compound **2** represent similar values of LDH leak in the culture medium, suggesting that both monomers and treated oligomers with compound **2** do not provoke a significant disturbance in cell membranes. However, once we assayed the supernatant obtained from nontreated oligomers, the LDH value increased, suggesting cell membrane damage and cytotoxic events.

## 3. Discussion

Tauopathies and Alzheimer’s disease are neurodegenerative disorders involving tau protein [2]. Tau is an unfolded protein linked to microtubules stabilization; however, it can form aggregates in specific brain regions provoking cellular damage and subsequent cognitive impairment [15]. The therapeutic strategy in clinical trials focuses on reducing MAPT expression, modulating post-translational modifications, preventing tau aggregation, immune clearance, and tau stabilizing agents [15]. Concerning tau inhibitors, methylene blue (Rember-TauRx, therapeutic) interferes with tau cysteine oxidation [16] and moved onto two clinical phase trials where it showed improved results on improving patient cognition by using 138 mg/day; however, no clinical effect was seen with a higher dose [17]. Moreover, a reduced form of methylene blue, LMTM (LMT-X, TRx0237), has had no positive outcomes in clinical trial phase three [18]. Interestingly, a compound named dimethyl fumarate serves as a treatment for multiple sclerosis and psoriasis [19,20].

**Scheme 1:**
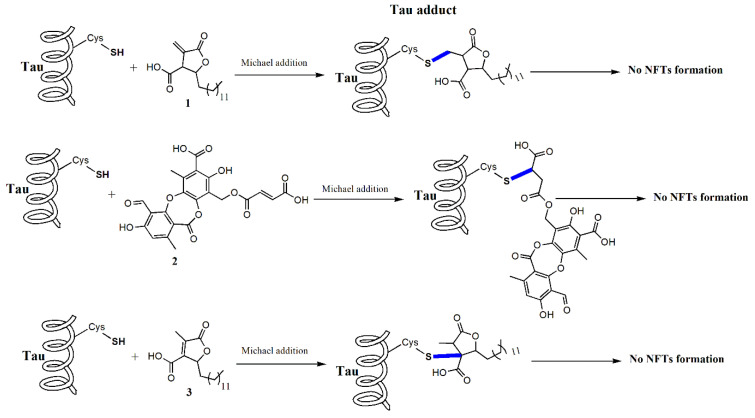
Covalent adduct formation between tau cysteine and compounds **1**, **2**, **3**. These compounds share an α, β unsaturated carbon that can interact with cysteines inside the microtubule-binding domain.

Thus, the exploration of inhibitors acting on cysteine through Michael’s addition increases as an alternative therapeutic option. In the present work, we isolated three compounds, **1**, **2**, and **3**, from Antarctic lichens, and all of them inhibited the progression of tau aggregates through the interaction with cysteine, as depicted in Scheme 1. These compounds share and α, β unsaturated carbonyl acting as a soft electrophile, making the β carbon susceptible to the attack of the soft nucleophile as cysteine. Then, we demonstrated that compound **2** could prevent changes in cell morphology monitored through a monoclonal antibody TU-01, whose epitope is located between amino acids 65 and 79 of alpha-tubulin, representing a stable form of microtubule dynamic. Interestingly, compound **2** can reduce LDH leaking, an enzyme related to cell membrane damage [21].

## 4. Materials and Methods

### 4.1. Plant Material

*Cladonia cariosa* and *Himantormia lugubris* were collected on Ardley Island, King George Island, the Antarctic region, in February 2021. Herbarium specimens were deposited in the Natural Product Laboratory of Chile University, Santiago, Chile, and Professor Alfredo Torres from the Austral University of Chile confirmed their identity.

### 4.2. Extraction and Isolation

*Cladonia cariosa* was first ground, then the samples (30 g) were placed in 200 mL of chloroform (3 times, 48 h/extraction). After filtration, the organic solutions were concentrated under reduced pressure to obtain a chloroformic extract (2 g). All chloroformic extract was dissolved in a minimum volume of boiling dichloromethane. The solution was allowed to cool to crystallize the fumarprotocetraric acid **2** (100 mg). The di-chloromethane solution was then concentrated (1.5 g) and submitted to column chromatography (CC) on silica gel 60 (63–200 µm, 200 g, column length 70 cm, i.d. 4 cm) and eluted with CHCl_3_/MeOH mixtures (200 mL each) of increasing polarity (9:1 up to 1:9) and MeOH. The eluates were monitored and pooled according to TLC analysis and then concentrated under reduced pressure to produce the isolation of usnic acid (250 mg) and protolichesterinic acid **1** (15 mg), as described [22].

*Himantormia lugubris* (20 g) was dried, powdered, and extracted with dichloromethane (3 × 100 mL, 48 h/extraction). The organic solutions were filtered and concentrated under reduced pressure, yielding 1.5 g of extract. The DCM extract was dissolved in methanol and subjected to Sephadex LH-20 using mobile phase MeOH. Some 70 fractions (15 mL) were monitored by TLC and combined to give two fractions, A-B. Fraction A (1.0 g) was chromatographed on silica gel (150 g, 63–200 µm) using n-hexane/EtOAc mixtures (0 up to 100%) to yield 120 mg of usnic acid and 20 mg of lichesterinic acid **3**, as described [23]. Fraction B (0.5 g) was chromatographed on silica gel (SiO_2_, 63–200 µm) using DCM/MeOH mixtures (0 up to 100%) and giving barbatolic acid (400 mg).

### 4.3. Protein Purification

Tau fragment 4R (htau244-372) was amplified by using the plasmid for htau40 as a template. The PCR amplified sequence was subcloned into a pET-28a vector (Novagen) to produce a His-tagged protein. The recombinant fragment 4R was expressed in Escherichia coli strain BL21 (DE3), as described [24]. LB medium containing kanamycin was inoculated with a stationary overnight culture. The culture was grown at 37 °C to OD 600 of 0.5–0.6, and protein expression was induced by adding 1 mM IPTG for 4 h. The cells were pelleted and sonicated. Recombinant tau was purified via ProPac IMAC 10 (Thermo Fisher Scientific, Waltham, MA, USA) using a gradient consisting of 10–200 mM imidazole, 20 mM Na_2_HPO_4_, and 500 mM NaCl. The purity of the protein was verified on a Coomassie brilliant blue-stained SDS-polyacrylamide gel. The protein was concentrated and stored at −80 °C until use. The concentration of purified 4R was determined using the extinction coefficient at 280 nm (1520 M^−1^ cm^−1^).

### 4.4. Thioflavin T Assay

The ThT fluorescence was done as described [24]. Briefly, to examine the inhibition of tau aggregation, the total volume of the reaction mixture was 100 μL, which included tau-heparin 4:1, 20 μM 4R: 5 μM heparin in 100 mM sodium acetate, and pH 7.0 in the presence or not of compound **2**. After 48 h of incubation, 100 μL of a 25 μM of ThT was added to the solution and incubated for 1 h at room temperature before fluorescence reading. Then, fluorescence was measured in a Biotek H1 multimode reader (Biotek Instruments, Winooski, VT, USA) with an excitation wavelength at 440 nm and emission wavelength at 485 nm in a 96-well plate, as described. Each experiment was done at least in triplicate, and background fluorescence was subtracted as needed.

### 4.5. Atomic Force Images

All atomic force images were obtained in a Nanoscope IIIa, in a fluid cell using the tapping mode through a SiN cantilever, whose stiffness constant ranges from 0.12 to 0.15 N/m. The tapping mode provides the phase information, making it possible to distinguish between the sample elements and establishing their presence or absence. In addition, AFM images in the tapping mode allow for determining the membrane surface’s topographic details, verifying its thickness using a cross-section obtained from the topographic image.

### 4.6. Maleimide Labeling

Protein samples were reduced with TCEP (tris(2-carboxyethyl) phosphine) by using a 10 M excess (TCEP 400 µM:4R 40 µM). Then, the mix was incubated for 2 h at 37 °C and eluted by using an Amicon Ultra centrifugal filter unit at 3500 rpm for 5 min at 4 °C (Merck Millipore, Darmstadt, Germany). After that, the protein was incubated with the fluorophore Alexa 488 Maleimide molar ratio (2:1) for 2 h at 30 °C. The excess of the fluorophore was eluted as described before. The induction of the aggregation of the protein labeled was done as described [14,25]. Briefly, tau protein was induced by adding a molar excess of heparin (10:40 µM) for 4 h. After that, samples were read in a Biotek H1 multimode reader in a 96-black plate flat bottom. The Ex/Em was set at 493/516 nm. Additionally, these samples were used for TIRFM experiments.

### 4.7. Total Internal Reflections Fluorescence Microscopy (TIRFM)

Samples were analyzed in a microscope Olympus IX81 linked to a color camera PL-B778F (Pixelink, ON, Canada). Briefly, samples were diluted at 1:10 in ultrapure water. Then, 5 µL were placed over the Menzel-Glaser coverslips (1.5 × 24 × 60 mm) over Ig TIRF (TIRF LAB). The laser, 465 nm, was turned on linked to a side-end excitation light launcher (TIRF LAB) to excite the fluorophore attached to the 4R (maleimide Alexa 488, Thermo Fisher Scientific, Waltham, MA, USA). Then, we selected the proper filter (FITC/EGFP/Fluo 3/DiO Acridine Orange(+DNA)). The samples were irradiated, acquiring image scanning of the surface of the coverslips by using the software Pixelink Capture OEMa (exposure 250 ms and gain 8 db).

### 4.8. Cells Culture, Immunofluorescence, and LDH Assay

N2a neuroblastoma cells were cultured at 37 °C in a humidified atmosphere at 5% CO_2_, using MMM Group^®^ Stove, Standard CO2 Cell 190, in DMEM D1152 plus F-12 medium supplemented with 10% fetal bovine serum (FBS) (Biowest, Nuaillé - France), 100 μg/mL ampicillin, and 100 μg/mL streptomycin, in T25 cell culture flasks. The cells were cultured and differentiated in Petri dishes, 35/10 mm over coverslips, then FBS was added at 2.5% plus retinoic acid of 10 μM for five days, replacing the medium culture every 48 h for neurite growth. Treated oligomers and oligomers were added on day four for 24 h. After that, cells were fixed for immunofluorescence and the supernatant recovered for LDH measurement. Cells previously incubated with either tau oligomers treated or oligomers alone were transferred to glass coverslips. The cells were then washed twice with 1X PBS buffer (137 mM NaCl, 10 mM Na_2_HPO_4_, 2.7 mM KCl, 1.76 mM KH_2_PO_4_), fixed for 30 min with 4% paraformaldehyde at room temperature, washed three times with PBS, and permeabilized for 10 min with Triton X100 0.5% (Sigma-Aldrich, St. Louis, MO, USA) diluted in PBS. Finally, coverslips were washed three times with PBS. After that, the cells were incubated with 1% BSA (Sigma-Aldrich St. Louis, MO, USA) for 30 min at room temperature. Subsequently, samples were incubated at 37 °C for one hour with the supernatant of α tubulin monoclonal TU-01 [26,27], diluted in blocking solution 0.1% BSA, and washed three times in PBS. After that, cells were incubated with Alexa Fluor 488 Goat anti-Mouse IgG (Thermofisher) for 1 h at room temperature and washed three times in PBS. Subsequently, the cells were mounted in 4′,6-diamidino-2-phenylindole (Vectashield, vector, Vectashield, Vector, Burlingame, CA, USA). Samples were analyzed with a BX61 microscope (Olympus, Tokyo, Japan). The LDH assay was performed as described with slight modifications [21]. The enzymatic activity was measured in a Mindray BA 88A semiauto chemistry analyzer (Mindray, Gurugram, Haryana, India) at 340 nm. The working solution was prepared with 2 mL of solution 1 (substrate → NADH or 8 mmol/L), plus 8 mL of solution 2 (Buffer Tris pH 7.0 50 mmol/L, Pyruvate 1.5 mmol/L). For the reaction, 10 μL of cell supernatant after exposure of lysis buffer (50 mM Tris-HCl (pH 7.4), 150 mM NaCl, 1% Triton X-100, and 5 mM EDTA), oligomers treated, and oligomers alone were loaded into 1 mL of working solution and measured in a spectrophotometer at 37 °C, at a wavelength of 340 nm for 180 s.

### 4.9. Data Analysis

Data analysis was performed using GraphPad Prism 6.0. Data are presented as mean ± SD and analyzed using one-way ANOVA. The analysis of TIRFM images was performed by using MATLAB and FIJI software. The experiments were done in triplicate.

## Data Availability

Data of the compounds are available from the authors.

## Supplementary Materials

The following are available online. Figures S1 and S2: Secondary metabolites isolated from lichens, and NMR; Figure S3: Histogram of TIRFM images; Figure S4: Thioflavin T assay; Figure S5: Histogram of AFM phase images; Figure S6: Tau filaments PDB 6tjx.1.

## Sample Availability

Samples of the compounds are not available from the authors.
