# Supplementary material for: The Fumarprotocetraric Acid Inhibits Tau Covalently, Avoiding Cytotoxicity of Aggregates in Cells
Source: Molecules. 2021 Jun 21;26(12):3760. doi: 10.3390/molecules26123760 (PMC8234475; doi:10.3390/molecules26123760)
Supplement: Supplementary file 1 [file molecules-26-03760-s001.zip › molecules-1221234-SI.pdf]

Supplementary material:

The fumarprotocetraric acid inhibits tau covalently, avoiding cytotoxicity of aggregates in cells

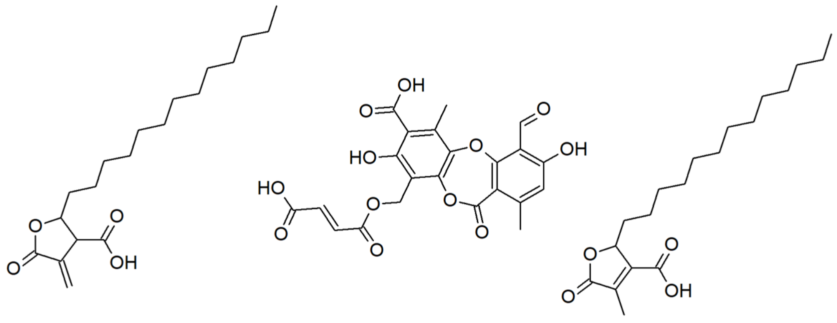

1 2 3

Figure S1. Secondary metabolites isolated from Antarctic lichens, (**1**) protolichesterinic acid, (**2**) fumarprotocetraric acid, (**3**) lichesterinic acid.

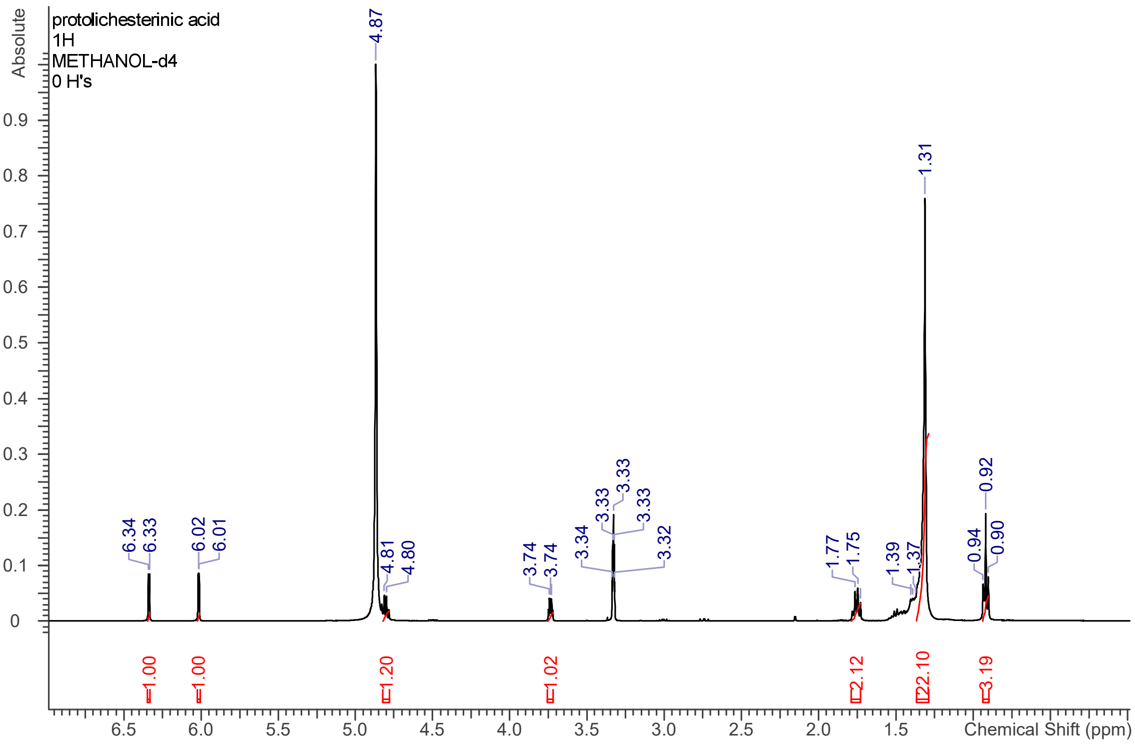
protolichesterinic acid
1H
METHANOL-d4
0 H's
Absolute
Chemical Shift (ppm)

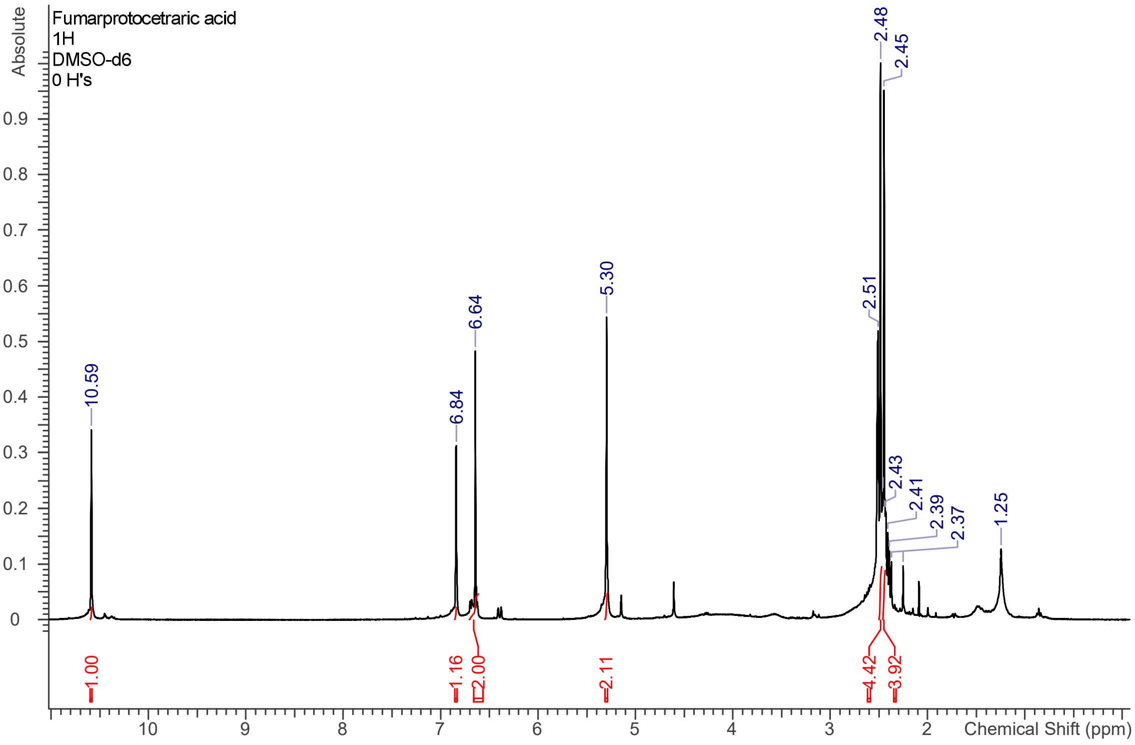
Fumarprotocetraric acid
1H
DMSO-d6
0 H's
Absolute
Chemical Shift (ppm)

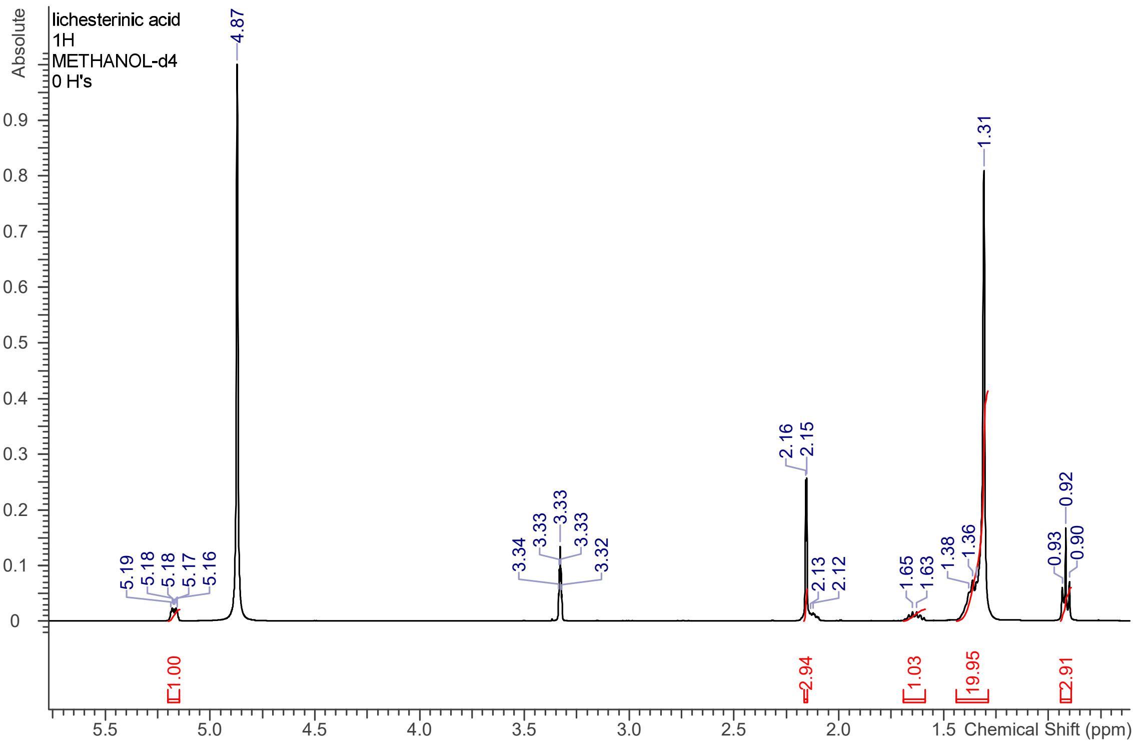

Figure S2. NMR of compound protolichesterinic acid (**1**), fumarprotocetraric acid (**2**), and lichesterinic acid (**3**) isolated from Antartic lichens.

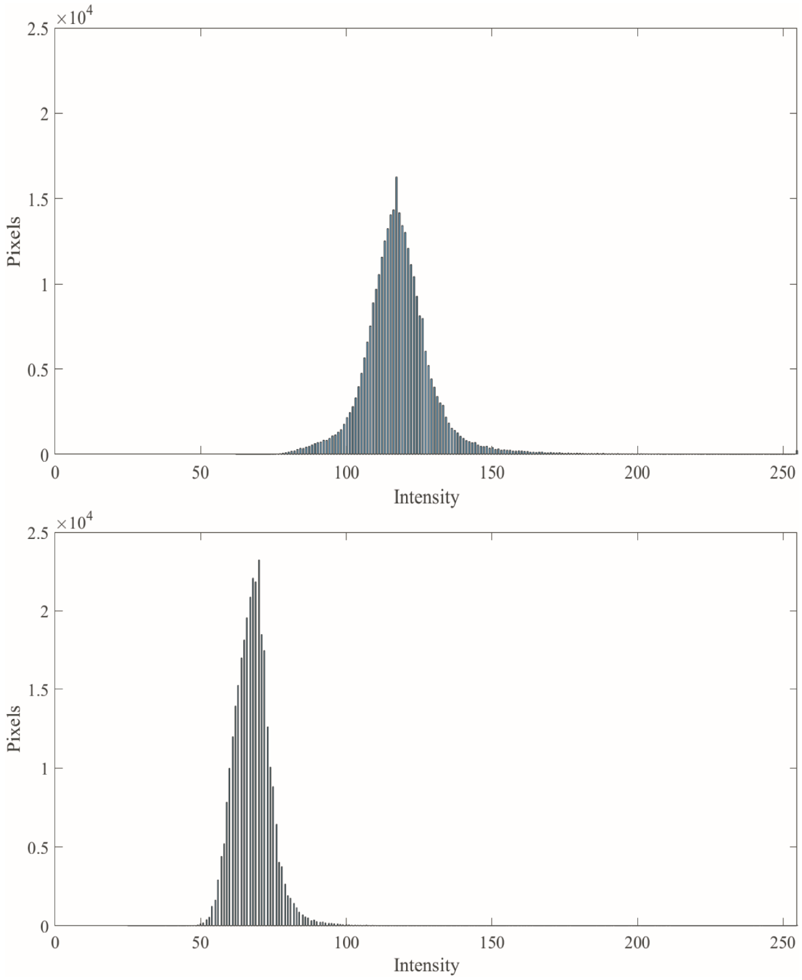

Figure S3. Upper panel, illuminated fluorophore Alexa 488 quantification of aggregation control samples. Lower panel, illuminated fluorophore Alexa 488 quantification of aggregation control samples treated with compound **2.** Data analyzed performed through pixel quantification in MATLAB software.

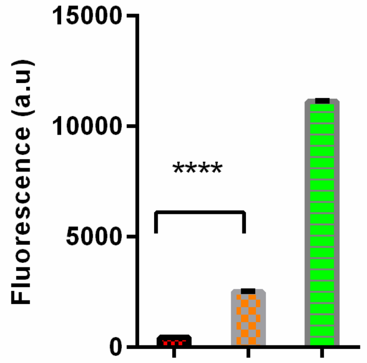

Figure S4. Thioflavin T assay at 48 hs of tau alone or in the presence of compound **2**, time 0 corresponds to monomeric tau. Data analyzed through One way Anova with Dunnett´s multiple comparison test *p<0.05*. The red, orange, and green fluorescent bars represent tau monomer, tau aggregates in the presence of **2,** and tau aggregates at 48 hs.

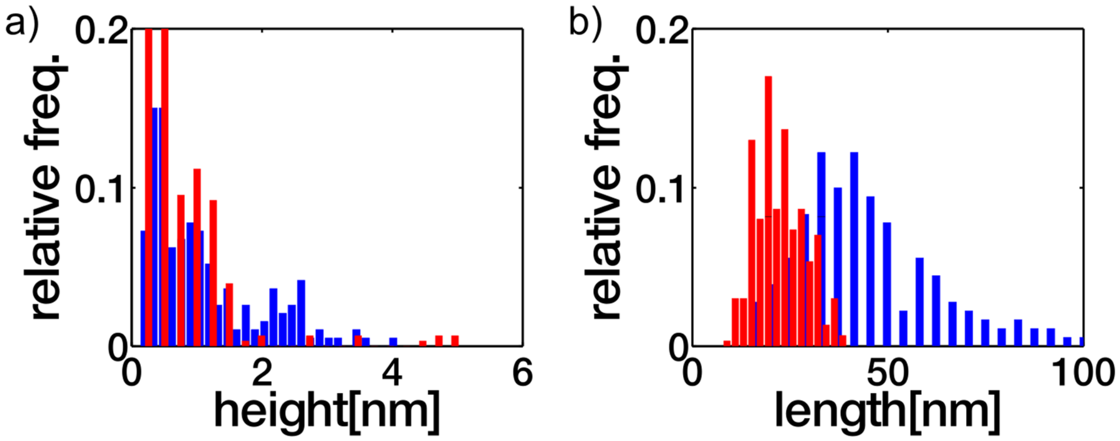

Figure S5. Histogram analysis of aggregates samples over HOPG and samples treated with compound **2.** Blue bars represent aggregates, and red bars represent tau aggregated treated with compound **2,** respectively.

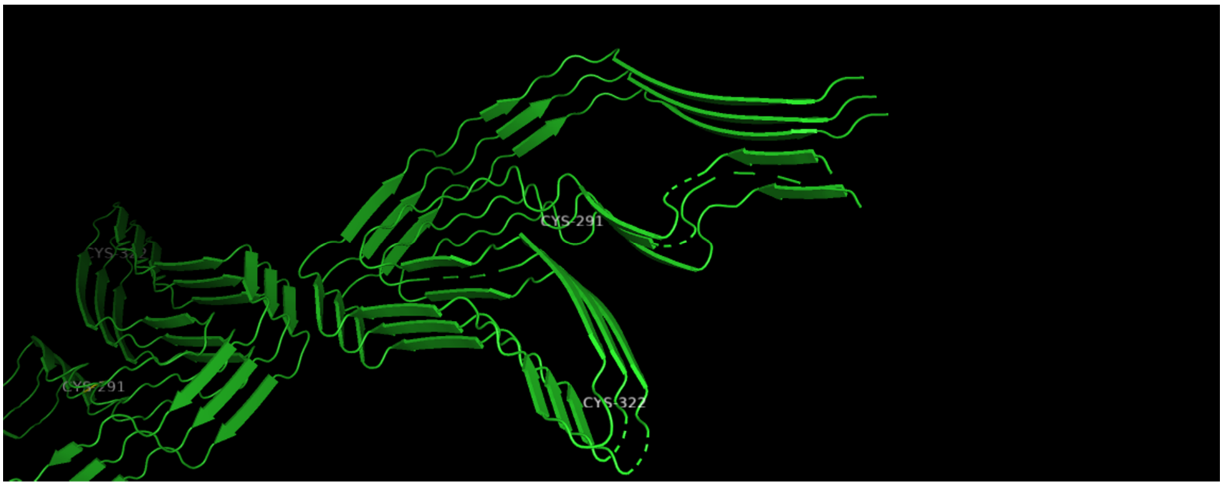

Figure S6. Tau filaments (PDB 6tjx.1) depicting Cysteines 291 and 322 involved in tau filament assembly. The depiction was done by using Pymol.
